# Supplementary material for: Serratiopeptidase Affects the Physiology of Pseudomonas aeruginosa Isolates from Cystic Fibrosis Patients
Source: Int J Mol Sci. 2022 Oct 20;23(20):12645. doi: 10.3390/ijms232012645 (PMC9604282; doi:10.3390/ijms232012645)
Supplement: Supplementary file 1 [file ijms-23-12645-s001.zip › ijms-1909912-supplementary.pdf]

Supplementary Material

Table S1. Antimicrobial profile of bacterial strains [36] and antimicrobial activity of SPEP.

Bacterial Strain	CAR	PTC	AM	QUIN	MB	CEF	COL	SPEP
PA14	S	S	S	S	S	S	S	>10000 U/ml
23P	R	S	R	R	S	R	S	>10000 U/ml
27P	S	S	S	S	S	S	S	>10000 U/ml
28P	S	S	S	S	S	S	S	>10000 U/ml
30P	S	S	S	S	I	S	S	>10000 U/ml

CAR: Carbapenems; MP: Meropenem; IP: Imipenem; PTC: Piperacillin/tazobactam; AM: Aminoglycosides; QUIN: Quinolones; CI: Ciprofoxacin; LE: Levofoxacin; MB: Monobactam; CEF: Cephalosporins; COL: Colistin; R: Resistant; S: Susceptible; I: Intermediate.

Figure S2.

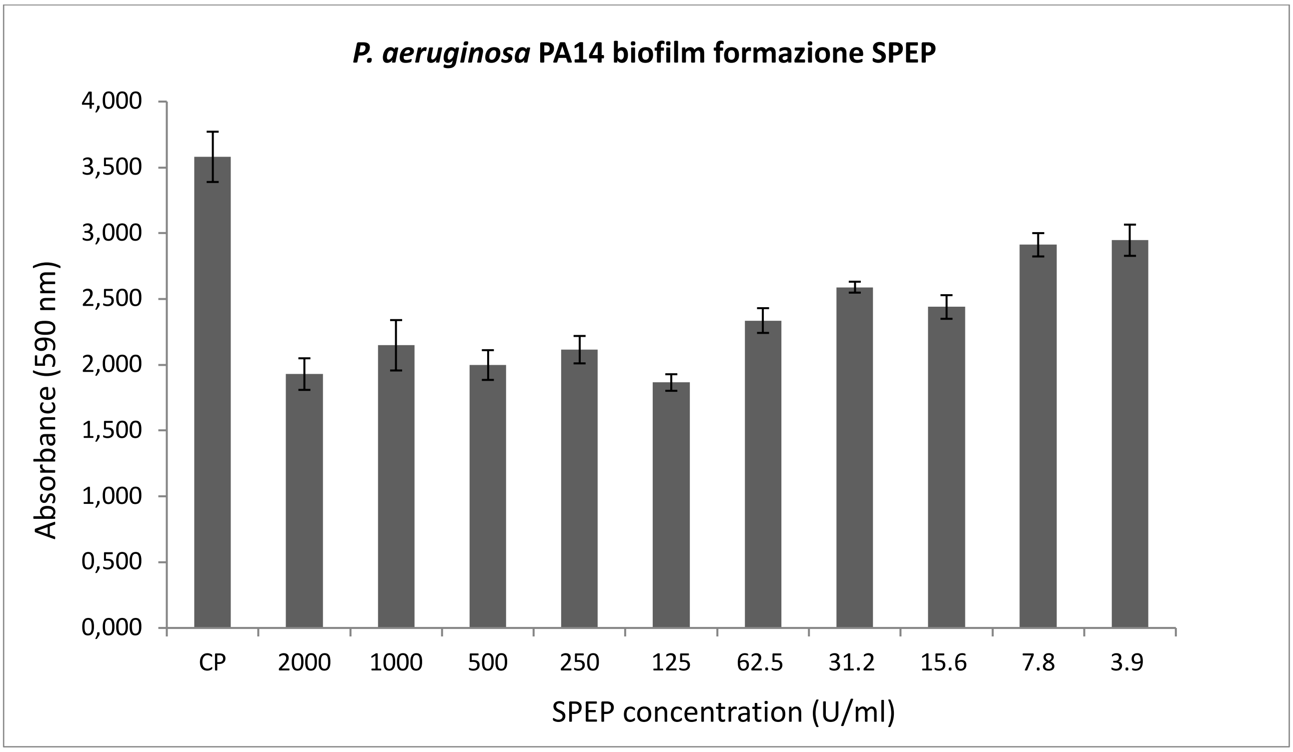

Figure S1. A dose-depending effect of SPEP on *P. aeruginosa* PA14 biofilm formation starting from a concentration of 2000 U/ml. Results were expressed as OD at 590 nm. Data reported are representative of three independent experiments. Error bars indicated the standard deviations of three measurements.

Figure S2.

A

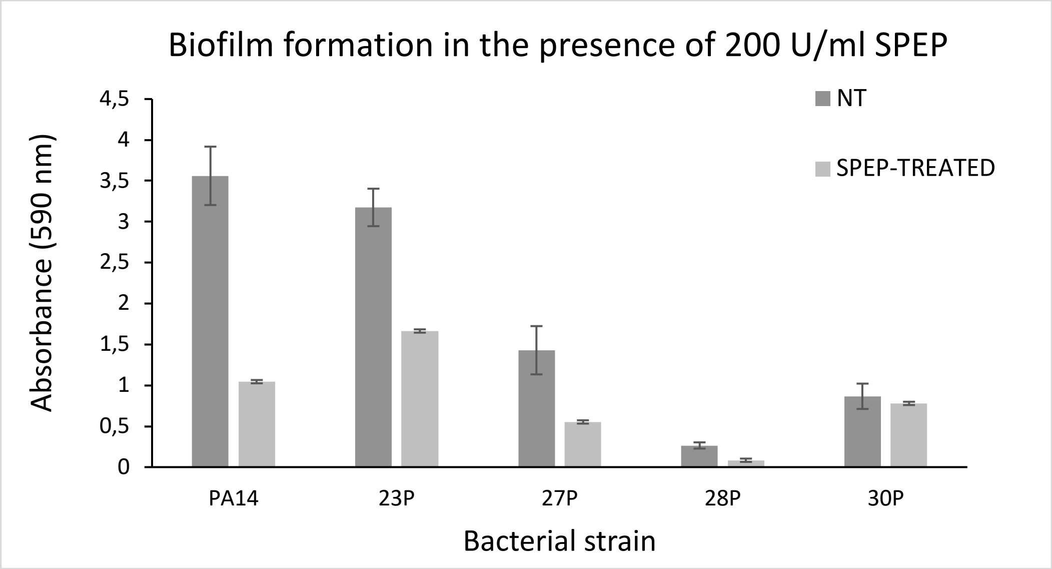

B

PA14

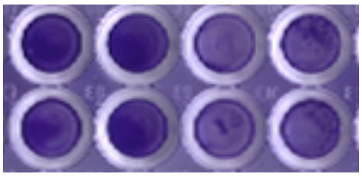

23P

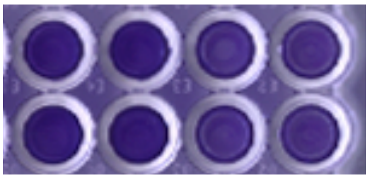

27P

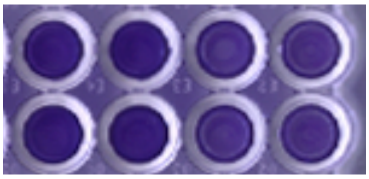

28P

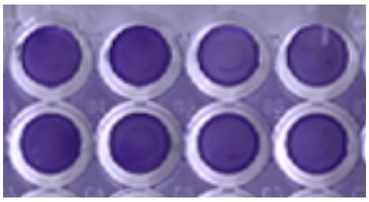

30P

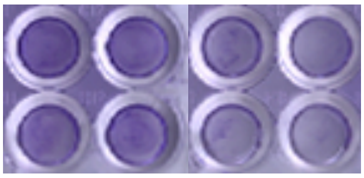

C

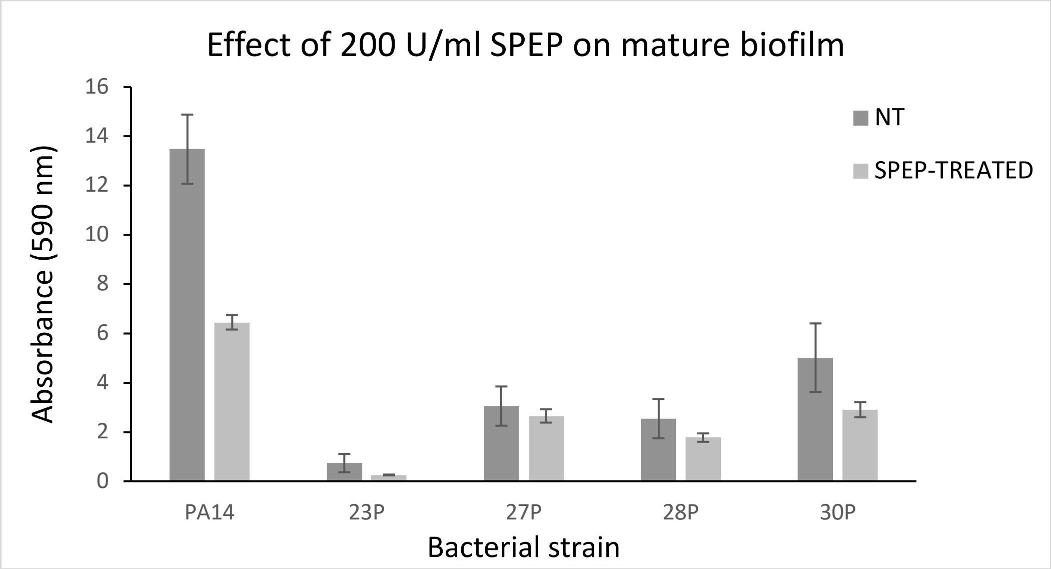

D

PA14

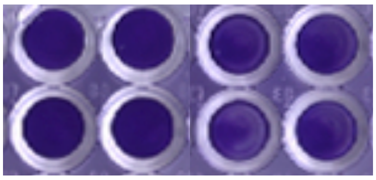

23P

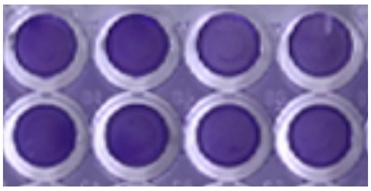

27P

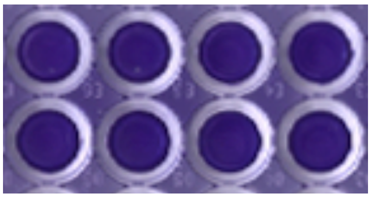

28P

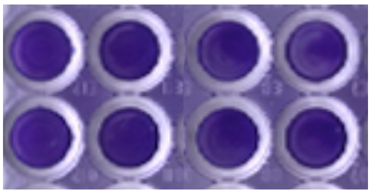

30P

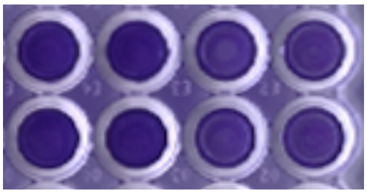

Figure S2. Effect of SPEP on biofilm formation of different clinical and reference strains. Panel A: Effect of SPEP on biofilm formation. In the ordinate axis is reported the absorbance at 590 nm. Each data point is composed of 4 independent experiments, each performed at least in 3-replicates. Panel B: Crystal violet coloration of biofilm formation of different clinical and reference strains in 96-wells. For each image, the first four wells represent the untreated samples, while the second four are SPEP-treated ones. Panel C: Effect of SPEP on 24 h mature biofilm. In the ordinate axis is reported the percentage of residual biofilm. Each data point is composed of 4 independent experiments, each performed at least in 3-replicates. Panel D: Crystal violet coloration of mature biofilm formation of different clinical and reference strains in 96-wells. For each image, the first four wells represent the untreated samples, while the second four are SPEP-treated ones.

Figure S3.

A

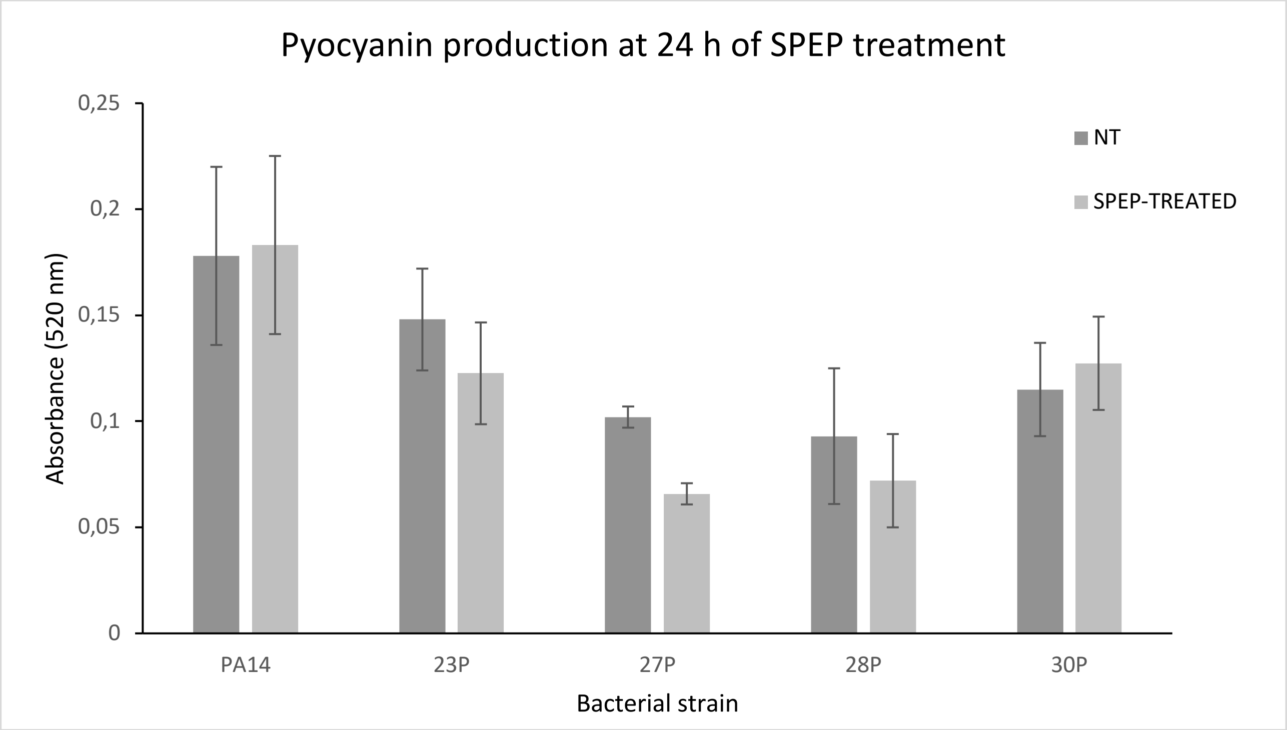
Pyocyanin production at 24 h of SPEP treatment
NT
SPEP-TREATED
Absorbance (520 nm)
0,25
0,2
0,15
0,1
0,05
0
PA14
23P
27P
28P
30P
Bacterial strain

B

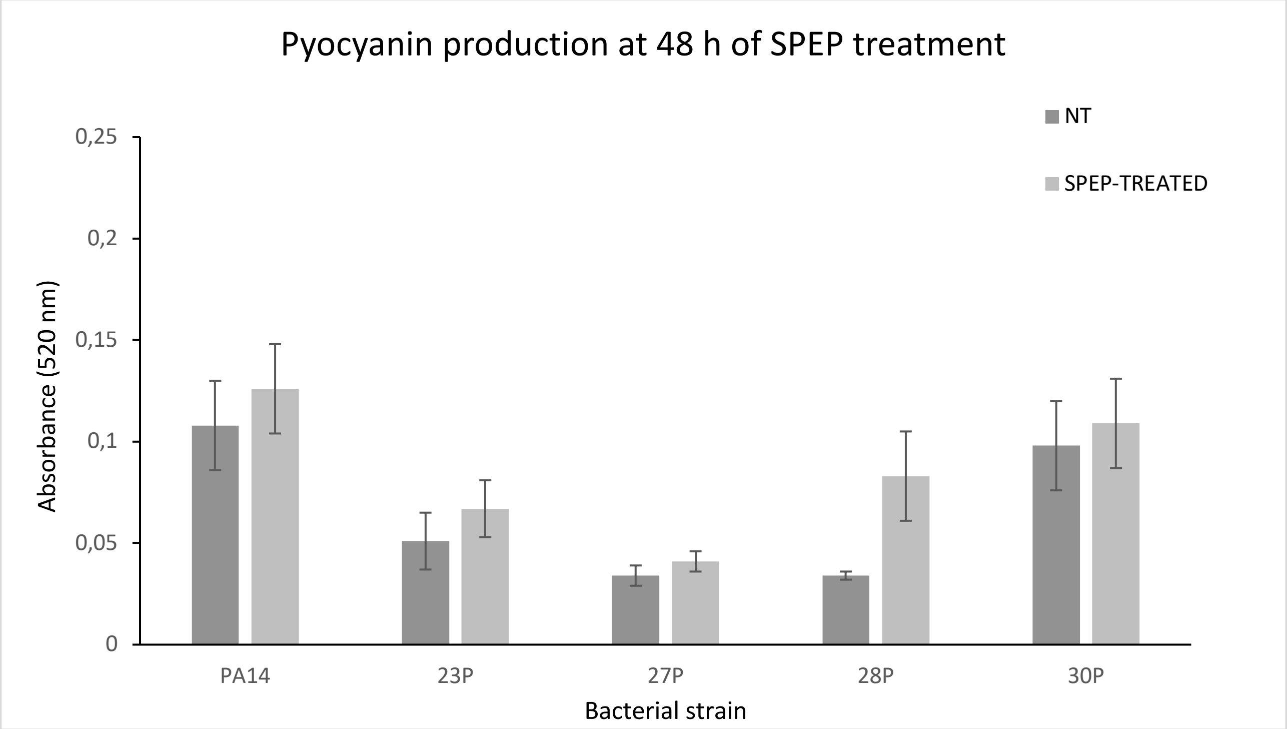
Pyocyanin production at 48 h of SPEP treatment
NT
SPEP-TREATED
Absorbance (520 nm)
0,25
0,2
0,15
0,1
0,05
0
PA14
23P
27P
28P
30P
Bacterial strain

C

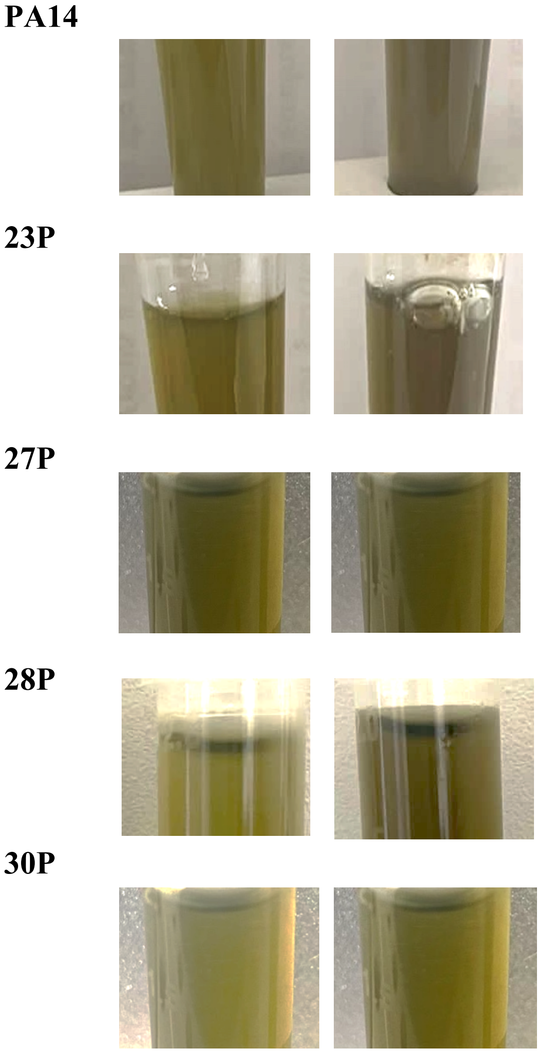

Figure S3. Effect of SPEP on pyocyanin produced after 24 (A) and 48 h (B) of growth. Data are reported as absorbance at 520 nm after pyocyanin extraction. Each data point is composed of 4 independent experiments, each performed in at least 3 replicates. Panel C: Images of SPEP effect (in the right images) on pyocyanin production of *P. aeruginosa* after 48 h of incubation.

Figure S4.

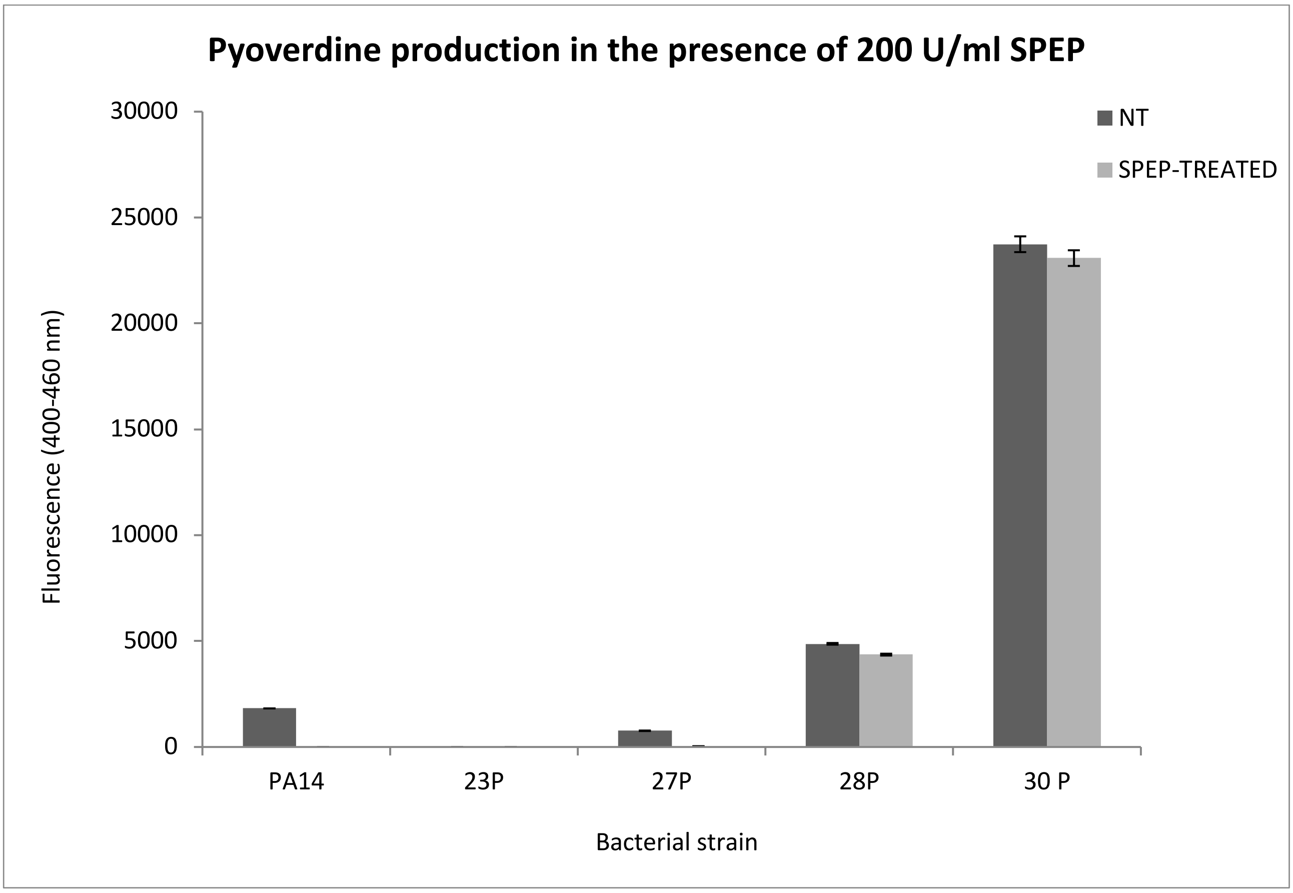

Figure S4. Effect of SPEP on pyoverdine produced after 48 h of growth. Data are reported as fluorescence recorded at 400 nm excitation 460 nm emission wavelengths. Each data point is composed of 4 independent experiments, each performed in at least 3-replicates. Error bars indicated the standard deviations of all the measurements.

Figure S5.

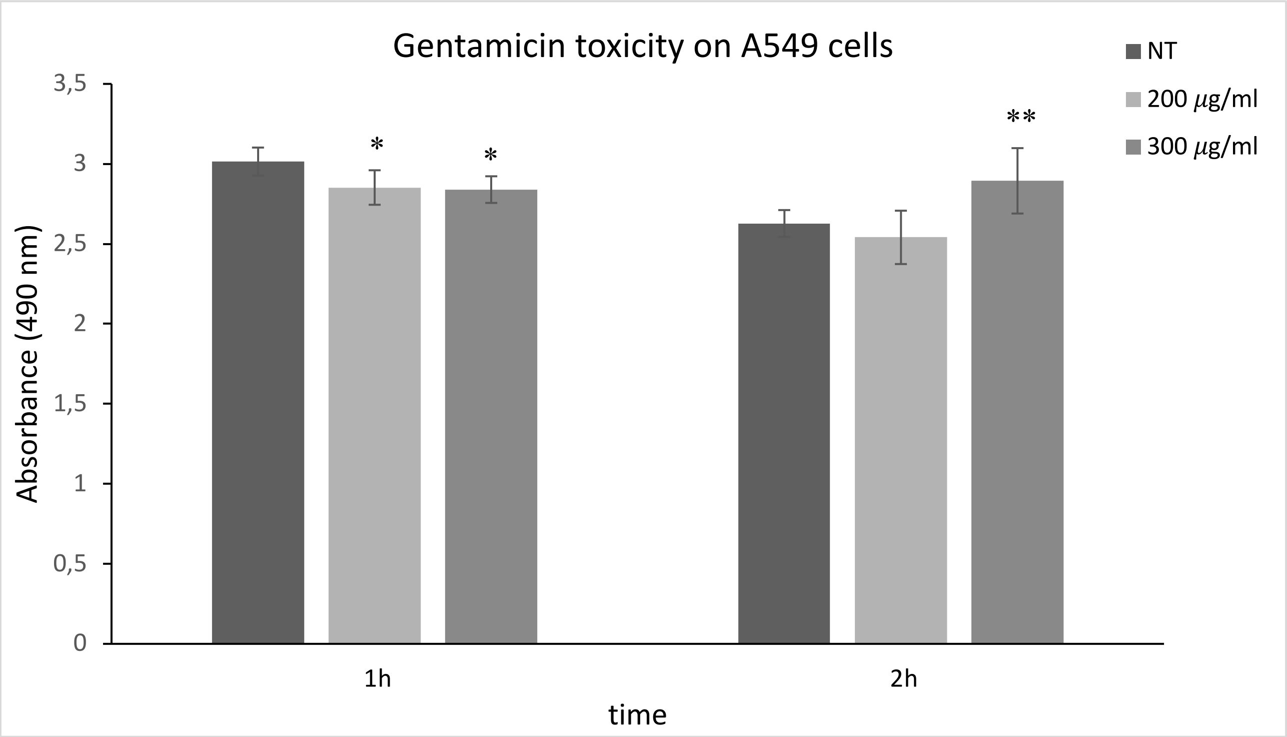

Figure S5. Effect of gentamicin on A549 immortalized cell lines. Dose-response plot of cells after 1 and 2 h incubation with increasing concentrations (200–300 μg/ml) of molecules. Cell viability was assessed by the MTT assay and expressed as described in the Materials and methods section. Values are given as means ± SD (n = 6); * indicates $p < 0.05$, ** indicates < 0.01with respect to control cells.
